# Case report: Successful treatment of refractory immune thrombocytopenia in chronic lymphocytic leukaemia with venetoclax monotherapy

**DOI:** 10.3389/fonc.2023.1260003

**Published:** 2023-10-18

**Authors:** Timothy Woo, Matthew Carter, George Follows, Piers EM. Patten

**Affiliations:** ^1^ Department of Haematology, King’s College Hospital National Health Service (NHS) Foundation Trust, London, United Kingdom; ^2^ Department of Haematology, Addenbrooke’s Hospital, Cambridge University Hospitals, Cambridge, United Kingdom; ^3^ Comprehensive Cancer Centre, School of Cancer and Pharmaceutical Medicine, King’s College London, London, United Kingdom

**Keywords:** chronic lymphocytic leukaemia, immune thrombocytopenia, refractory, venetoclax, monotherapy

## Abstract

In chronic lymphocytic leukaemia (CLL), immune dysregulation is common and can manifest as immune thrombocytopenia (ITP). Corticosteroids are the mainstay for front-line management of CLL-associated ITP. Therapy refractoriness represents a clinical challenge and is an indication to commence CLL-directed treatment, historically with anti-CD20 antibody-based chemoimmunotherapy. There is a small but growing body of evidence supporting the use of Bruton’s tyrosine kinase (BTK) inhibitors in this setting, but not the B-cell lymphoma-2 inhibitor, venetoclax. Here, we describe two cases of refractory ITP in patients with CLL who successfully achieved and sustained complete remission with fixed-duration venetoclax monotherapy. Responses were rapid and durable and not explained by the concomitant use of an anti-CD20 antibody. This supports a dual role for single-agent venetoclax in managing active CLL and associated ITP as an alternative to BTK inhibitors and anti-CD20 monoclonals.

## Introduction

Chronic lymphocytic leukaemia (CLL) is a heterogeneous malignancy characterised by the clonal proliferation of mature CD5^+^ B cells within lymphoid tissues. In CLL, disease-intrinsic and therapy-related immune dysregulation is common, clinically presenting as recurrent infections and less frequently as autoimmune phenomena. Autoimmune haemolytic anaemia (AIHA) and immune thrombocytopenia (ITP) occur in up to 10% and 5% of patients, respectively (1). The pathologic basis for autoimmunity is not fully elucidated, although CLL-mediated loss of self-tolerance is postulated to be contributory (2).

Corticosteroids are the mainstay for front-line management of autoimmune cytopenias in quiescent CLL (3). Adjunctive thrombopoietin (TPO) analogue and intravenous immunoglobulin (IVIG) also have a role in the context of ITP. Treatment refractoriness to initial measures signifies the need to initiate CLL-directed therapy (4). There is a small but growing body of evidence supporting the efficacy of small-molecule inhibitors in treating CLL-associated autoimmune cytopenias (3, 5).

Here, we present two cases of treatment-refractory ITP in CLL, which successfully responded to monotherapy with the B-cell lymphoma-2 (BCL-2) inhibitor, venetoclax.

## Case 1

A 76-year-old man was admitted to hospital in December 2020 following the development of a petechial rash and oral blood blisters. He had a background of Binet stage A CLL with a 13q deletion (type I) and no adverse features such as trisomy 12, 17p deletion, or 11q deletion. This was diagnosed in February 2015, and he was managed conservatively with a watchful waiting approach.

**Figure 1:**
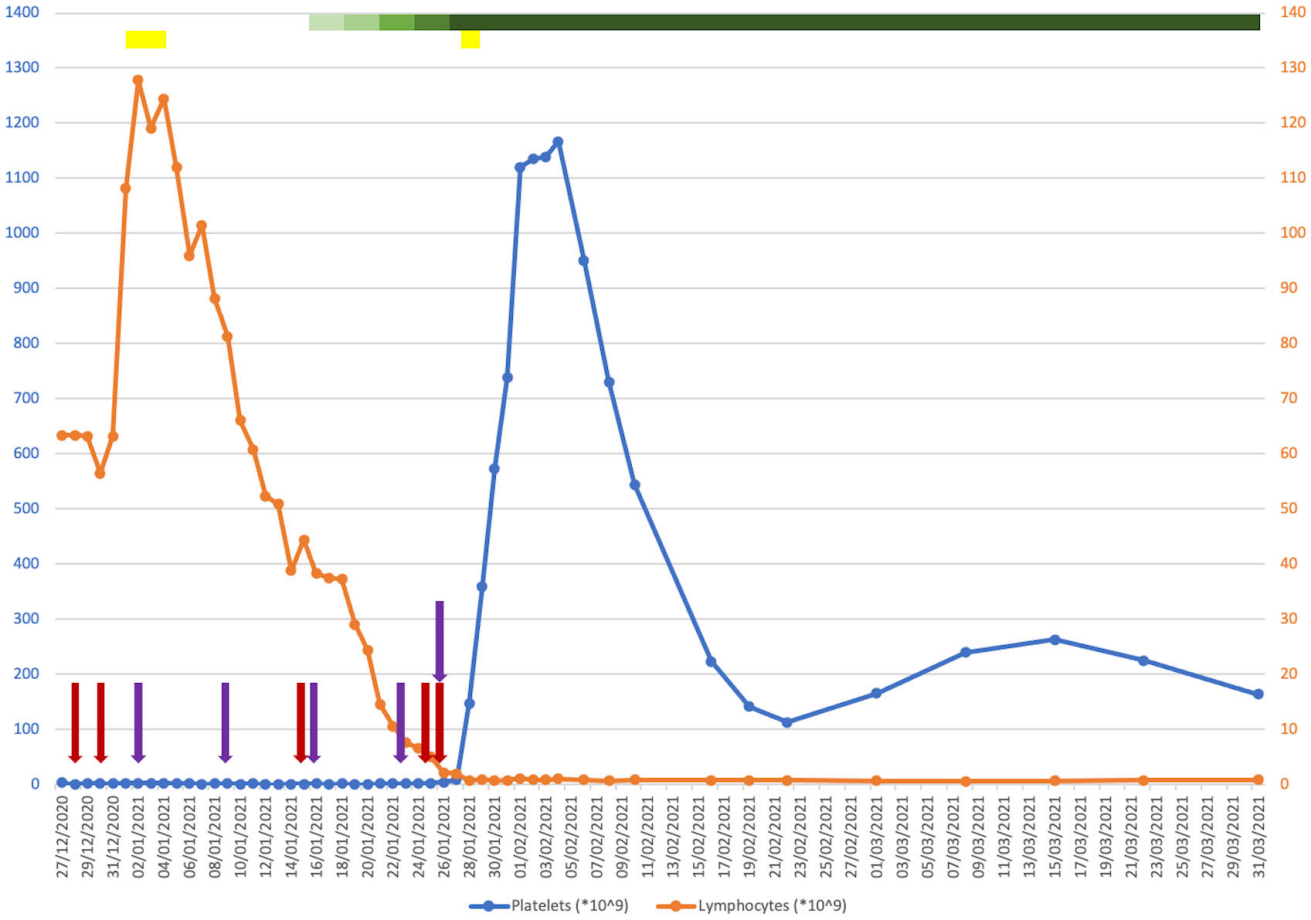
Plot of platelet (blue) and lymphocyte (orange) counts against time from presentation to 1 month post-discharge. Coloured bars indicate drugs that were given for a course of more than one consecutive day. Coloured arrows indicate drugs that were given on a one-off basis on discrete days. Red, intravenous immunoglobulin (IVIG); purple, romiplostim; yellow, corticosteroids; green (with a graded change in the colouring showing when the dosage was ramped up), venetoclax.

His platelet count at presentation was 4 * 10^9^/L (150–400 * 10^9^/L), having fallen from 173 * 10^9^/L when last measured in September (**Figure 1**). His haemoglobin and lymphocyte counts were 122 g/L (130–180 g/L) and 63.4 * 10^9^/L (1.0–4.8 * 10^9^/L), respectively, both stable at baseline. His coagulation profile was within normal limits. A peripheral blood film confirmed true thrombocytopenia with normal red cell morphology and lymphocytosis. There were no other bleeding symptoms at that time. There was no evidence of splenomegaly or active infection.

He was commenced on IVIG 1 g/kg followed by a 4-day course of dexamethasone 20 mg. He experienced a short-lived episode of epistaxis in the interim. Due to a lack of response, he was started on romiplostim initially at 1 μg/kg, which continued on a once-weekly basis. An examination of his bone marrow revealed infiltration by small monotonous lymphoid cells, trilineage haematopoiesis with plentiful megakaryocytes, and no increase in immature precursors. He was increasingly symptomatic from a bleeding perspective with the development of a haematoma post-bone marrow biopsy, rectal bleeding from existing haemorrhoids and melaena secondary to gastritis, and a small extra-axial haemorrhage. His haemoglobin downtrended as a result of a nadir of 58 g/L, requiring frequent red cell transfusion support. He was refractory to platelet transfusions. He received further doses of IVIG and a short course of methylprednisolone to no effect.

A decision was made to commence single-agent venetoclax at 20 mg, which was ramped up every 3 days without evidence of tumour lysis syndrome. Due to a concomitant infection with COVID-19 during his inpatient stay, obinutuzumab was withheld. Within 2 weeks of starting venetoclax and shortly after reaching daily dosing of 400 mg, a dramatic increment in his platelet count was observed, coinciding with a sustained downtrend in his lymphocyte count to below the reference range. He remained on venetoclax while romiplostim was discontinued. He was successfully discharged from hospital in mid-February 2021. His platelet counts were monitored closely in an outpatient setting over the following months, attaining satisfactory stability around an average of 100–120 * 10^9^/L. He was given romiplostim at approximately once-weekly intervals for 3 months post-discharge, which was ceased thereafter. Later that year, he completed a course of obinutuzumab for the treatment of his CLL, and venetoclax was discontinued in January 2022. His measurable residual disease in peripheral blood was negative at the end of treatment. He has since reverted to being managed with a watchful waiting approach, which maintains to this day.

## Case 2

A second case is of an 84-year-old man who presented initially in August 2020 with a 2-week history of a skin rash, blood blisters in the mouth, and gum bleeding. He presented due to worsening gum bleeding and, on examination, was found to have a petechial rash, subconjunctival haemorrhage, and wet mucosal bleeding in the mouth.

He had a background of atrial fibrillation, a cardiac pacemaker, coronary artery disease with eight previous cardiac stents, and hypertension. Flow cytometry 18 months previously had shown monoclonal B-cell lymphocytosis with a typical CLL phenotype. He took apixaban for thromboprophylaxis and aspirin given his cardiac disease history.

**Figure 2:**
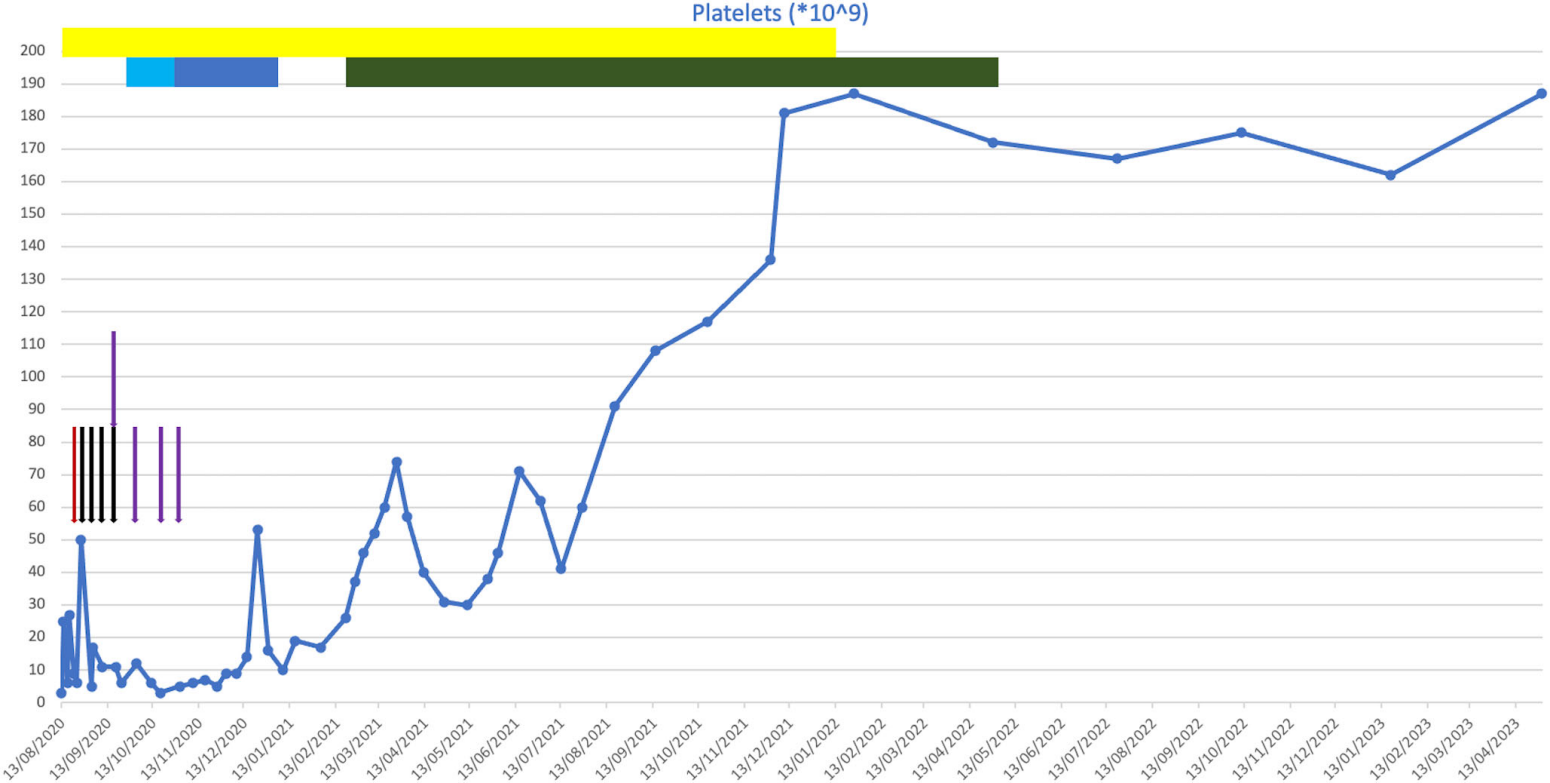
Plot of platelet counts against time from presentation to present day. Coloured bars indicate drugs that were given for a course of more than one consecutive day. Coloured arrows indicate drugs that were given on a one-off basis on discrete days. Red, intravenous immunoglobulin (IVIG); black, rituximab; purple, romiplostim; yellow, corticosteroids; light blue, chlorambucil; dark blue, cyclophosphamide; green, venetoclax.

At presentation, he was found to have a platelet count of 3 * 10^9^/L (**Figure 2**). A blood film showed a genuine thrombocytopenia with a monomorphic population of medium-sized lymphocytes. He had a bone marrow biopsy, which showed appearances consistent with high-volume bone marrow involvement, with >85% small B cells present with a CLL phenotype.

He was initially treated with a platelet transfusion and prednisolone at 1 mg/kg. His apixaban and aspirin were withheld. After 6 days of prednisolone, his platelet count had not improved (6 * 10^9^/L), and he required further platelet transfusion due to bleeding symptoms. He subsequently received treatment with IVIG (1 g/kg) a few days later due to intracranial bleeding following a fall.

As his platelet count did not improve with IVIG, he then went on to have treatment with rituximab and romiplostim. Shortly following this, he developed further wet mucosal bleeding and was treated with further IVIG. He continued on weekly rituximab for 4 weeks (the last dose was in September 2020) and was treated with romiplostim. Despite these treatments, his platelet count remained low at 26 * 10^9^/L. Owing to his frailty, frequent falls, and significant cardiovascular comorbidities, he was determined to be at high risk of complications from Bruton’s tyrosine kinase (BTK) inhibitor therapy. He was therefore treated with chlorambucil at 2 mg daily, which caused dizziness, and then followed this with oral cyclophosphamide. This was continued for 2 months, following which it was discontinued due to a lack of response.

Following this, he was initiated on venetoclax in February 2021. This led to an improvement in his platelet count, even at a low dose during the initial dose escalation period, which normalised soon after the initiation of venetoclax. He had no evidence of tumour lysis syndrome during this period. The dose was up-titrated to a maximum tolerated dose of 200 mg daily, which was continued until April 2022, at which point the patient developed mucositis leading to the venetoclax being held. His thrombocytopenia has remained in remission since cessation of the venetoclax, and he remains in clinical remission 13 months after completing venetoclax treatment. No additional anti-CD20 monoclonal was given following the commencement of venetoclax.

## Discussion

Consensus on the management approach of autoimmune cytopenias in CLL remains largely that of expert opinion and retrospective case studies. Corticosteroids are the mainstay of initial treatment, alongside adjunctive TPO analogue and IVIG in the context of ITP. Refractoriness to standard therapies represents a therapeutic challenge and is an indication to begin CLL-directed treatment. Historically, this would often constitute an anti-CD20 antibody-based chemoimmunotherapy regimen. Similarly, CD20 antagonism with rituximab is also considered beneficial in the treatment of idiopathic ITP (6).

Venetoclax is an inhibitor of the anti-apoptotic protein BCL-2, which is upregulated in CLL cells (7). It is increasingly adopted in routine practice for the management of active CLL in treatment-naïve and relapsed-refractory patients, usually as a fixed-duration therapy with an anti-CD20 antibody (i.e., obinutuzumab in the front-line or rituximab in the relapsed setting) in combination (8). There is a paucity of literature evaluating its role in the setting of autoimmune cytopenias in CLL (5). By contrast, the use of BTK inhibitors in this scenario is more prevalent. However, given that they require continuous administration until disease progression, it is unclear whether treatment cessation may precipitate relapse of the ITP. Moreover, the association of BTK inhibitors with cardiotoxic complications may preclude their selection in patients with concurrent cardiovascular comorbidities (9).

Here, we describe two cases of refractory ITP in patients with CLL who successfully achieved and sustained complete remission with venetoclax monotherapy. A striking feature of our two cases is the exquisite sensitivity both showed to the introduction of single-agent venetoclax. Responses were rapid and durable and not explained by the concomitant use of an anti-CD20 antibody. Considering the potent immunosuppressive properties of anti-CD20 agents, especially when given in conjunction with other therapies directed against CLL, deferring (as with our first case) or avoiding (as with our second case once venetoclax was initiated) their use in certain circumstances such as concomitant COVID-19—particularly taking into account the clinical vulnerability of patients with CLL towards infections—may be beneficial (10). We hypothesise that in our cases, the resolution of the autoimmune cytopenia was related to venetoclax-driven depletion or elimination of the CLL clone since platelet recovery coincided exactly with the time of development of peripheral blood lymphopenia. Nonetheless, other contributory immunomodulatory effects cannot be excluded, and this represents an important area for further study.

Three case reports have documented the use of venetoclax in CLL-associated ITP (11–13). Similar to the cases demonstrated here, in all three reports, the patients were refractory to multiple lines of treatment before attaining a response upon initiation of venetoclax, although one patient had received rituximab in combination and another relapsed 5 weeks after discontinuation of adjunctive prednisolone and romiplostim.

In this report, we highlight the ability of time-limited venetoclax monotherapy to achieve rapid and long-lasting remissions of CLL-associated ITP. This supports a dual role for venetoclax in managing active CLL and associated ITP as a single agent, and its utility should be considered early in patients unresponsive to front-line measures especially where BTK inhibitors may not be as appropriate.

## Data availability statement

The original contributions presented in the study are included in the article/supplementary material. Further inquiries can be directed to the corresponding author.

## Ethics statement

Written informed consent was obtained from the participant/patient(s) for the publication of this case report.

## Author contributions

TW: Data curation, Writing – original draft, Writing – review & editing. MC: Data curation, Writing – original draft, Writing – review & editing. GF: Writing – review & editing. PP: Conceptualization, Supervision, Writing – review & editing.
